# Graphene Oxide Incorporated Polysulfone Substrate for Flat Sheet Thin Film Nanocomposite Pressure Retarded Osmosis Membrane

**DOI:** 10.3390/membranes10120416

**Published:** 2020-12-11

**Authors:** Siti Nur Amirah Idris, Nora Jullok, Woei Jye Lau, Hui Lin Ong, Cheng-Di Dong

**Affiliations:** 1 Faculty of Chemical Engineering Technology, Universiti Malaysia Perlis, Kompleks Pusat Pengajian Jejawi 3, Kawasan Perindustrian Jejawi, Arau 02600, Malaysia; nuramie@gmail.com (S.N.A.I.); ong.huilin@gmail.com (H.L.O.); 2 Centre of Excellence for Biomass Utilization & Taiwan-Malaysia Innovation Centre for Clean Water and Sustainable Energy (WISE Center), Universiti Malaysia Perlis, Lot 17, Kompleks Pusat Pengajian Jejawi 2, Jejawi, Arau 02600, Malaysia; 3 Advanced Membrane Technology Research Centre, Universiti Teknologi Malaysia, UTM, Skudai Johor 81310, Malaysia; lwoeijye@utm.my; 4 Department of Marine Environmental Engineering, National Kaohsiung University of Science and Technology, 142, Hai-Chuan Road, Nan-Tzu District, Kaohsiung 81157, Taiwan; cddong@nkust.edu.tw

**Keywords:** graphene oxide, polysulfone, pressure retarded osmosis, thin film nanocomposite membrane, power density

## Abstract

This study focuses on the development of flat sheet thin film nanocomposite (TFN) pressure retarded osmosis (PRO) membranes for the enhancement of osmotic power generation by the incorporation of laboratory-synthesised graphene oxide (GO) into the polysulfone (PSf) polymer matrix. A series of membranes containing different weight percent of GO (0, 0.1, 0.25, 0.5 and 1.0 wt%) were fabricated via a phase inversion method with polyethylene glycol (PEG) as the pore forming agent. The results show that the TFN-0.25GO membrane has excellent water flux, salt reverse flux, high porosity and an enhanced microvoids morphology compared to the control membrane. The highest power density was achieved when TFN-0.25GO was used is 8.36 Wm^−2^ at pressure >15 bar. It was found that the incorporation of GO into the polymer matrix has significantly improved the intrinsic and mechanical properties of the membrane.

## 1. Introduction

Pressure retarded osmosis (PRO) is a promising source of renewable energy [1]. The PRO process involves a pressurised draw solution (DS), which is diluted by the water that permeates through a semipermeable membrane from a low salinity feed solution (FS). The energy generated from the osmosis process is then converted into mechanical energy and electricity using a hydro turbine and generator, respectively. Numerous new PRO membranes and processes have been developed, and the most critical features in PRO are balancing the membrane power density and generating sufficient applied pressure [2]. The general equation under ideal conditions, the theoretical water flux, *J_w_*, can be estimated using Equation (1):

*J_w_* = *A* (Δ*π* − Δ*P*) (1)

where *A* is water permeability, Δ*π* is the osmotic pressure difference and Δ*P* is the pressure difference across the membrane. An important parameter in PRO is power density, *W*, which can be calculated using Equation (2):

*W* = *J_w_*Δ*P* (2)

Under ideal conditions, where Δ*π* and *A* are constant, *W* production is given by:

*W* = *A* (Δ*π* − Δ*P*) Δ*P* (3)

It has been found that maximum *W* production is obtained when Δ*P =* Δ*π/2*. *W_max_* can be obtained by [3,4,5]:

*W_max_* = *A* (Δ*π^2^*/4) (4)

However, the actual PRO process often includes salt leakage, an internal concentration polarization (ICP) in the support layer of the membrane, and external concentration polarization (ECP), which occurs on the membrane’s surface. This occurrence reduces the mass transfer within the membrane, induces the pressure drop and eventually reduces the power production in PRO. Therefore, the fabrication of high-performance PRO membranes that can produce an experimental *W_max_* closer to the theoretical value has become very important.

Previous researchers focused on developing thin film composite via an interfacial polymerization (IP) process that gives a promising performance. Li et al. [6] fabricated thin film composite (TFC) polyetherimide membranes with three different substrate structures. They reported a high water permeability of 483 Lm^−2^h^−1^, a high porosity of 73.5% and a stable water flux value at a pressure of 17.2 bar. Zhang et al. [7] modified their fabricated TFC polyacrylonitrile membrane with the aid of pre-compression and alcohol as post-treatment. The membrane can withstand pressure up to 10 bar and achieved a power density of 2.6 Wm^−2^. Indeed, some researchers have recently reported the development of remarkably high performance TFC PRO membranes, but all are still in the laboratory scale [8,9,10]. To date, no commercial TFC PRO membrane can demonstrate a power density greater than 5 Wm^−2^. Thus, to further improve the performance of the flat sheet TFC PRO membrane, the strategy of modifying the membrane substrate should be further explored.

Incorporation of hydrophilic nanomaterials into the polymeric membranes (skin layer or/and substrate layer) led to a new generation of TFC membranes called thin film nanocomposite (TFN) membranes. This includes nanomaterials such as modified carbon nanotubes [11,12,13], covalent organic framework [14], zeolites [15,16] and graphene oxide (GO) [17,18]. Due to the enhanced hydrophilicity, structural parameter, water flux and mechanical properties that lowered the ICP phenomenon, an excellent TFC PRO performance was achieved [18].

Graphene and its derivatives (e.g., GO, reduced GO (rGO) and nanoporous graphene (NG)) have been explored by many researchers for their potential applications in various fields such as water purification [19], water desalination [20], gas separation [21], fuel cell and renewable energy system [22]. Graphene oxide has attracted tremendous attention due to its straightforward, scalable, high hydrophilicity and it contains abundant oxygenous functional groups including carboxyl, epoxy and hydroxyl groups. It also has high surface area, high tensile strength and high chemical stability [23,24,25,26]. Their excellent high surface-area-to-volume ratios would promote efficient interaction with the polymer matrix in a mixed matrix membrane (MMM) [27,28]. Park et al. [17] synthesised a TFN hollow fibre PRO membrane with optimum GO loading of 0.2 wt%. They achieved a high water flux of 43.74 Lm^−2^h^−1^, and the membrane withstood pressure up to 16.5 bar. The dual-layered TFN PRO membrane incorporated with 0.25 wt% GO, and 4 wt% halloysite nanotubes (HNT) developed by Lim et al. [18] exhibited higher power density, high mechanical strength and showed outstanding resistance to membrane fouling. However, there are a limited number of examples of flat sheet TFN PRO membranes incorporated with GO in the literature.

In this study, a series of flat sheet TFN membranes were fabricated with lab-synthesised GO incorporated in a polysulfone (PSf) polymer via mixed matrix process with polyamide (PA) layer by m-phenylenediamine (MPD) and 1,3,5-benzene-tricarbonyl trichloride (TMC) in n-hexane via IP. These membranes were designed to have improved structural properties and hydrophilicity to reduce the ICP phenomenon. The characteristics of the synthesised GO were examined by Scanning Electron Microscopy (SEM), X-Ray Diffraction (XRD), Fourier Transform Infrared Spectroscopy (FTIR) and Raman spectroscopy. Meanwhile, the characteristics and performance of the flat sheet TFN-GO PRO membranes were thoroughly investigated, including the morphology, functional groups, porosity, intrinsic properties, structural parameters and contact angles. Finally, the optimum wt% GO loading was determined through the intrinsic properties, forward osmosis (FO) and PRO performance.

## 2. Materials and Methods

### 2.1. Materials

Natural flakes graphite (average particles diameter 40 mm, 99.95% purity) was purchased from Sigma Aldrich, MO, USA. Polysulfone granules (PSf) (Udel^®^ P-1700 LCD, Mw 67,000 gmol^−1^) were purchased from Solvay Advanced Polymer, GA, USA. Polyethylene glycol (PEG; M_w_: 600 gmol^−1^) and potassium permanganate (KMnO_4_) were supplied from Merck, Darmstadt, Germany. The 1-methyl-2-pyrrolidinone (NMP, 99%) and m-Phenylenediamine (MPD, 99+ %) were used without any further purification from Acros Organics, NJ, USA. 1,3,5-benzene-tricarbonyl trichloride (TMC), sulphuric acid (H_2_SO_4_, 95–97%) and phosphoric acid (H_3_PO_4_, 98%) from Sigma Aldrich, MO, USA. Hydrogen peroxide (H_2_O_2_) and hydrochloric acid (HCl, 36.5–38.0%) were supplied by J. T. Baker, NJ, USA. The sodium chloride (NaCl, M_w_ 58.44 gmol^−1^) used in the preparation of the draw solution was purchased from HmbG^®^ Chemicals, Hamburg, Germany. Deionized water (Milli-Q) with a resistivity of 18 MΩ cm was used throughout the experiments.

### 2.2. Preparation of GO

Graphene oxide was synthesised by the thermal exfoliation of natural graphite flakes via the modified Hummer’s method [29]. First, 2.0 g of graphite flakes were added to 225 mL of sulphuric acid in a 500 mL two-necked round bottom flask and then stirred using a magnetic stirrer for 10 min. Next, 6.0 g of KMnO_4_ was slowly added to the solution and stirred at 50 °C for 10 h. Then, the mixture was diluted with 225 mL of DI water in an ice bath. The colour of the mixture became brilliant yellow. 5 mL of H_2_O_2_ was added and stirred for 30 min to remove the residual permanganate. The mixture was washed with 1 M HCl to remove the remaining manganese ions from the mixture, followed by centrifugation and washing with DI water. The final mixture was continuously washed with DI water and dried in a freeze dryer. The GO was then collected and stored at 4 °C for further use.

### 2.3. Characterisation of GO

The surface morphology of the synthesised GO was characterised by SU8010 SEM (Hitachi Tokyo, Japan) with an acceleration electron voltage of 15 kV. All samples were Pt-coated using an Ion Sputter ε-1030 (Hitachi, Tokyo, Japan) to increase the conductivity. Transmission electron microscopy (TEM) was performed using a JEM-2010F (JEOL, Tokyo, Japan). XRD patterns were recorded with a Bruker D8 Advance X-ray diffractometer (Bruker, MA, USA) with Ni-filtered Cu Kα radiation (λ = 1.5406 Å) operated at a generator voltage of 40 kV and an emission current of 40 mA. Raman spectra were collected using a Bruker Senterra micro-Raman spectrometer equipped with a laser power of 5 W operating at a wavelength of 532 nm. FTIR spectra were obtained using a Horiba FT-720 spectrometer (Horiba, Kyoto, Japan) at a resolution of 2 cm^−1^ in KBr pellets.

### 2.4. Preparation of TFN-GO Membrane

The TFN-GO membranes were fabricated by the non-solvent induced phase separation (NIPS) method. The varied GO loadings (0, 0.1, 0.25, 0.5 and 1.0 wt%) were homogeneously dispersed in NMP via ultra-sonication for 10 h at 40 ± 1 °C. The 18 wt% PSf were added to the solution and was mixed using an IKA^®^ Roller 6 (IKA^®^-Werke GmbH, Staufen, Germany) basic until the solution became homogeneous. All the dope solutions were placed in a vacuum desiccator to remove air and moisture before the fabrication of the membranes. The bubble-free dope solution was spread on a glass plate using an automated casting machine. The homogenous dope solution was evenly cast across the glass plate at a controlled thickness of 200 µm using a stainless-steel casting knife. The glass plate was then dipped immediately into a non-solvent bath containing deionized (DI) water and kept there for 5 min to allow the phase-inversion immersion precipitation process to take place. The polymeric film (i.e., the nascent membrane) was then kept under running DI water for 5 min to remove the residual solvent and stored in DI water before the interfacial polymerization is carried out.

The polyamide (PA) dense active layer on top of the substrate was formed via interfacial polymerization (IP). The top surface of the support layer was soaked in a 2 wt% MPD solution for 3 min. After drying at room temperature for 30 s, 0.1 wt% of TMC in n-hexane was poured onto the support layer for 1 min. Then, the membrane was dried in a vacuum oven at 60 °C for 30 min. The prepared TFN flat sheet PA/PSf-PEG membrane was rinsed thoroughly with DI water to remove the residual monomers and kept in DI water before use. The prepared TFN-GO membranes were denoted as TFN-0GO, TFN-0.1GO, TFN-0.25GO, TFN-0.5GO and TFN-1.0GO reflecting the GO loaded in the dope solution.

### 2.5. Characterisation of the TFN-GO Membrane

#### 2.5.1. Morphology, Surface Roughness, Functional Group and Contact Angle

The morphology of the cross-section and active layer of the TFN-GO membranes was observed with SEM. The membranes were first dried in an oven for 24 h to remove moisture. The membranes were then fractured using liquid nitrogen to get a consistent and clean cut of the membrane cross-section for imaging. All the samples were sputter-coated with platinum before observation.

Atomic force microscopy in tapping mode was used to analyse the surface roughness of the membrane and to render three-dimensional images of the surface. Small parts of the membranes with approximately 1 cm^−2^ were cut and glued on a glass substrate.

The functional groups of the membranes were examined by FTIR in attenuated total reflectance (ATR) mode (Spectrum 65, PerkinElmer Inc., CA, USA). The spectrum for each sample was scanned 32 times from 450 cm^−1^ to 4000 cm^−1^ with 4 cm^−1^ resolutions.

The contact angle (CA) of the membranes was measured with a contact angle instrument (OCA 15Pro, Dataphysics Instruments Gmbh, Filderstadt, Germany). Ten measurements were carried out at random locations on the active layer of the membrane to yield the average value.

#### 2.5.2. Reverse Osmosis Test for Intrinsic Transport Properties of TFN-GO Membranes

The intrinsic parameters of the membranes were evaluated with a Sterlitech HP4750 high pressure stirred cell at, with an effective membrane area of 14.6 cm^2^. The water permeability, *A*, was measured using DI water as a feed solution and pressurised at different transmembrane pressures (10, 15 and 20 bar). The value of *A* was calculated using Equation (5):

(5) $$A=\frac{∆V_{a}}{∆t_{a}\times A_{m}\times ∆P}$$

where Δ*V_a_* is the permeate volume, Δ*t_a_* is the predetermined time, *A_m_* is the effective area of the membrane sample and Δ*P* is the transmembrane pressure difference.

The salt permeability, *B*, was determined using 1000 ppm of NaCl as a feed solution under a hydraulic pressure of 10 bar. The salt rejection, *R* was calculated by the following equation:

(6) $$R=\left(1-\frac{C_{p}}{C_{f}}\right)\times 100\%$$

where *C_f_* and *C_p_* are the permeate and feed salt concentrations. The value of *B* was calculated using Equation (7) where Δ*P* and Δ*π* are the transmembrane hydraulic and osmotic pressure differences, respectively.

(7) $$B=A\times \frac{1}{R}\times \left(∆P-∆\pi \right)$$

The structural parameter, *S*, of the membrane was calculated by the classical flux-fitting method using Equation (8) where *D* is the solute diffusion coefficient, *π_draw_* and *π_feed_* are the osmotic pressure of the draw and feed solutions, and *J_w_* is the flux under FO mode [30,31].

(8) $$S=\frac{D}{J_{w}}\ln\left[\frac{B+\left(A\times \pi_{draw}\right)}{B+J_{w}+\left(A\times \pi_{feed}\right)}\right]$$

#### 2.5.3. Porosity and Average Pore Size of the TFN-GO Membranes

The porosity, *ε*, of the membranes was measured from the difference between the wet and dry weights. The membranes stored in DI water were weighed using an electronic balance after excess water was removed with tissue paper. Then, the wet membranes were dried in a vacuum oven for 24 h at a temperature of 60 °C and weighed in the dry state. The porosity was calculated by Equation (9):

(9) $$\varepsilon =\frac{m_{w}-m_{d}}{\rho \times T\times A_{m}}$$

where *m_w_* is the wet weight, *m_d_* is the dry weight, *ρ* is the density of DI water, *T* is the membrane thickness and *A_m_* is the effective area of the membrane sample.

The average pore size, *r_m_*, was determined based on pure water permeability, *PWP*, and porosity data using the Gerout–Elford–Ferry equation (Equation (10)) [30]:

(10) $$r_{m}=\sqrt{8\times \frac{\left(2.9-1.75\varepsilon \right)\times \eta \times T\times PWP}{\varepsilon }}$$

where *η* and *T* are the viscosity of DI water and the thickness of the membrane sample, respectively. The *PWP* of the membranes was determined using the same high pressure stirred cell used to test *A* and *B* at 1 bar.

### 2.6. FO and PRO Membrane Performance

The FO performance was evaluated using a cross-flow filtration setup with an effective membrane area of 0.0042 m^2^. The DS and FS, of 1 M NaCl and DI water, respectively, were circulated concurrently through a membrane cell at a fixed flow rate of 1.50 L/min. The weight changes of DS for FO and FS for PRO were recorded by a data-logging balance every 2 s for 5 h after the system became stable. The NaCl concentration changes in the feed and draw solutions were recorded by a conductivity meter (Sensor Direct 150, Lovibond, FL, USA). The FO performance was tested with the membrane having an active layer facing feed solution (AL-FS) orientation, while the PRO was tested with an active layer facing draw solution (AL-DS) orientation. The pressure draw solution in PRO was controlled over a wide range of pressure (>15 bar), and all the experiments were carried out at 24 ± 1 °C.

The water flux, *J_w_*, was determined by the volume change in a DS (FO) and FS (PRO), Δ*V* in a predetermined time interval, Δ*t* using Equation (11):

(11) $$J_{w}=\frac{∆V}{A_{m}\times ∆t}$$

where *A_m_* is the effective membrane area of the membrane.

The salt reverse flux, *J_s_*, was calculated using Equation (12):

(12) $$J_{s}=\frac{C_{t}\times V_{t}-C_{0}\times V_{0}}{A_{m}\times {∆}_{t}}$$

where *C_t_* and *C*_0_ are the initial and final concentrations of the feed solution and *V_t_* and *V*_0_ are the initial and final volumes of the feed solution, respectively.

The power density, *W*, was determined by the product of the water flux, *J_w_*, and the applied operating pressure, Δ*P*.

(13) $$W=J_{w}\times ∆P$$

## 3. Results and Discussion

### 3.1. Characterisation of GO

**Figure 1:**
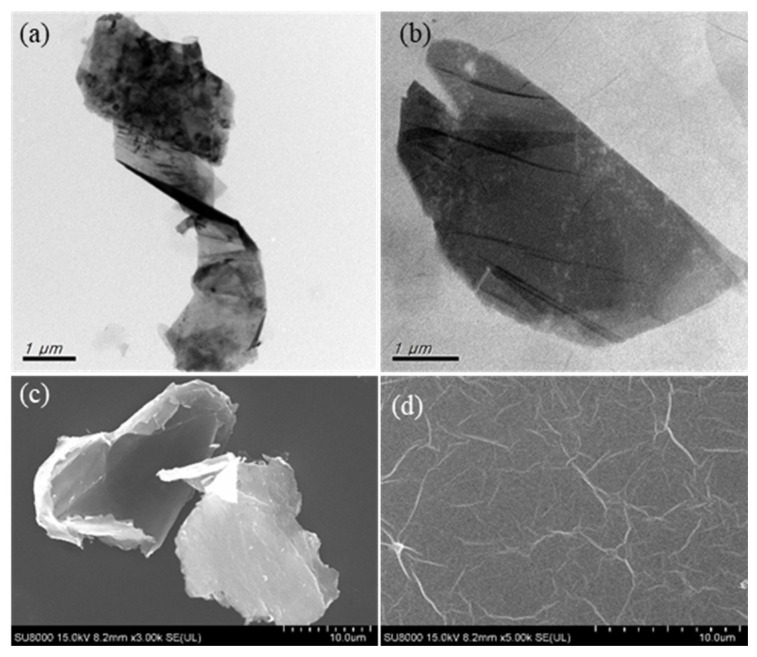
TEM images of (**a**) graphite and (**b**) synthesised graphene oxide (GO). SEM images of (**c**) graphite and (**d**) synthesised GO.

TEM and SEM are important techniques for studying microstructure at the nanoscale in great detail. Figure 1 shows TEM and SEM images of the graphite and the synthesised GO. The images for graphite flakes (Figure 1a,c) show the twisted platelet-like wrinkled microstructure. The wrinkled edge with a smooth surface was observed on synthesised GO nano-sheets (Figure 1b). Meanwhile, the SEM magnified surface area image of synthesised GO confirmed the presence of single layer nano-sheet agglomerates with typical wrinkled structure. These results agree with other reports in the literature [28].

**Figure 2:**
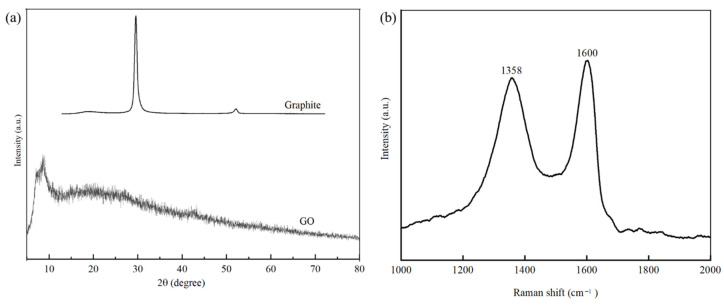
(**a**) XRD pattern of graphite and synthesised GO. (**b**) Raman shift of synthesised GO.

The successful synthesis of GO nano-sheets was confirmed by XRD, Raman and FTIR characterisations. The XRD patterns of graphite and synthesised GO are shown in Figure 2a. The sharp diffraction peak observed at 2θ = 10.9° is the characteristic peak corresponding to GO [32]. Sharp branches appear in the GO XRD pattern imply the presence of more oxygenated functional groups in GO after the chemical exfoliation of the graphite via Hummer’s method [33]. In addition, the disappearance of the diffraction peak of graphite at 2θ = 26.1°, which is characteristic of non-oxidised graphite, indicates the complete formation of GO [34,35].

The typical Raman shift for synthesised GO shown in Figure 2b has two significant peaks; the D peak at 1600 cm^−1^ and the G peak at 1358 cm^−1^ caused by the stretching vibration of sp^2^, particularly the distortion of the carbon atom [36]. According to the Raman shift of the synthesised GO, the ratio of the D and G peak (ID/IG) of 0.89 is close to values from the literature using the same method to synthesised GO [37,38]. The ID/IG ratio has been widely used to determine the disorder and degree of graphitisation of carbonaceous materials.

**Figure 3:**
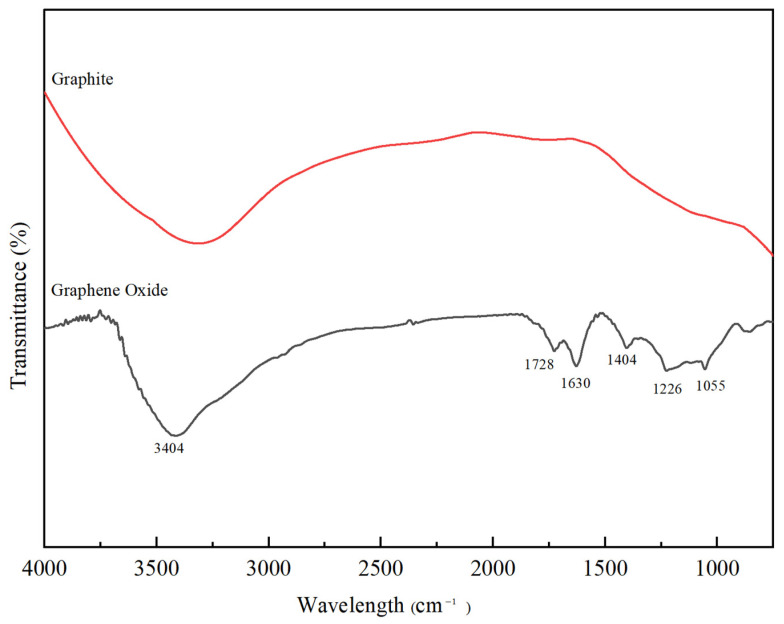
FTIR spectra of graphite and synthesised GO.

Figure 3 shows the FTIR spectrum of graphite and synthesised GO. The strong peak at 3404 cm^−1^ indicates the O-H stretching vibration from water molecules adsorbed on GO [39]. The peaks at 1728 cm^−1^ and 1226 cm^−1^ are due to the C=O and C−O stretching in the –COOH groups. The peak at 1630 cm^−1^ is attributed to a C=C stretching vibration. The peak at 1404 cm^−1^ is assigned to the stretching vibration of O−H in C−OH groups [36]. The peaks around 1055 cm^−1^ can be ascribed to the C−O−C stretching vibrations of the epoxy group [40]. The presence of an intense O-H group together with epoxy, carboxyl and hydroxyl groups led to the conclusion that the synthesised GO has strong hydrophilicity [41]. Meanwhile, there are no significant peaks observed in the graphite compared to the GO, indicating that the oxidation process was successful.

### 3.2. Characterisation of TFN-GO Membranes

**Figure 4:**
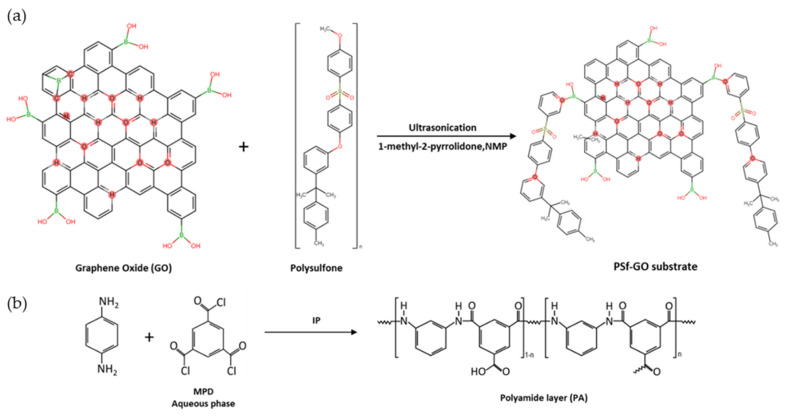
Schematic illustration of the interaction of (**a**) GO with PSf in 1-methyl-2-pyrrolidinone (NMP) and (**b**) polyamide (PA) layer via the interfacial polymerization (IP) process on top of the membrane substrates.

The synthesised GO was dispersed in NMP with PSf via sonication until the dope solution became homogenous. The membrane substrates were fabricated via the NIPS method before the interaction of MPD and TMC in n-hexane was introduced to the top layer of the membrane substrates during the IP process. The possible reaction mechanism is displayed in Figure 4.

**Figure 5:**
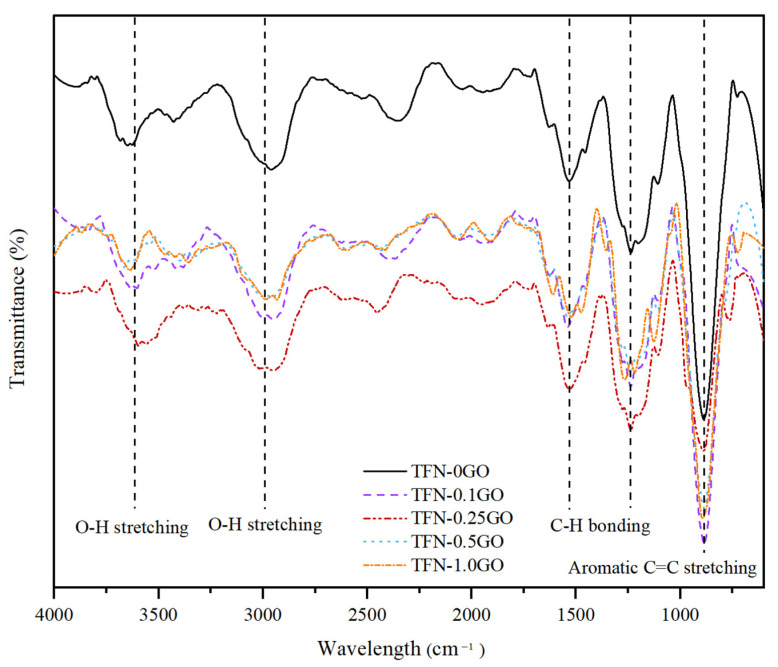
FTIR spectra of all thin film nanocomposite (TFN)-GO membranes.

This reaction mechanism can be confirmed by the FTIR characterisation of the TFN-GO membranes. Figure 5 shows the FTIR spectra of all the TFN-GO membranes: the transmittance decreased with the addition of GO. Interestingly, the TFN-0.25GO transmittance was more pronounced compared to the other membranes. The broad peaks of the O-H stretching vibration at 3600 cm^−1^ and 2990 cm^−1^, together with the peak associated with the epoxy group C−C at 1248 cm^−1^, clearly confirm the dispersion of GO in the polysulfone matrix [25,42]. Peaks of PSf were also observed at 1253 cm^−1^, due to the O=S=O asymmetric stretching of the sulfonate group [32].

On the other hand, the peaks at 1516cm^−1^ and 1483cm^−1^ represent the C=C bond also exist in the PSf repeat chain [43]. The strong peaks at 1488 cm^−1^ and 1588.5 cm^−1^ represent the amide-II aromatic in-plane ring C-H bending and indicate the successful formation of the PA layer [44,45]. Furthermore, other transmittance peaks of PA were observed at 876 cm^−1^, 800 cm^−1^ and 797 cm^−1^ that are attributed to the aromatic C=C stretching and C−O stretching of carboxylic acid groups that formed from the hydrolysis reaction of acid chloride groups of TMC in the crosslinking PA process [46,47].

**Figure 6:**
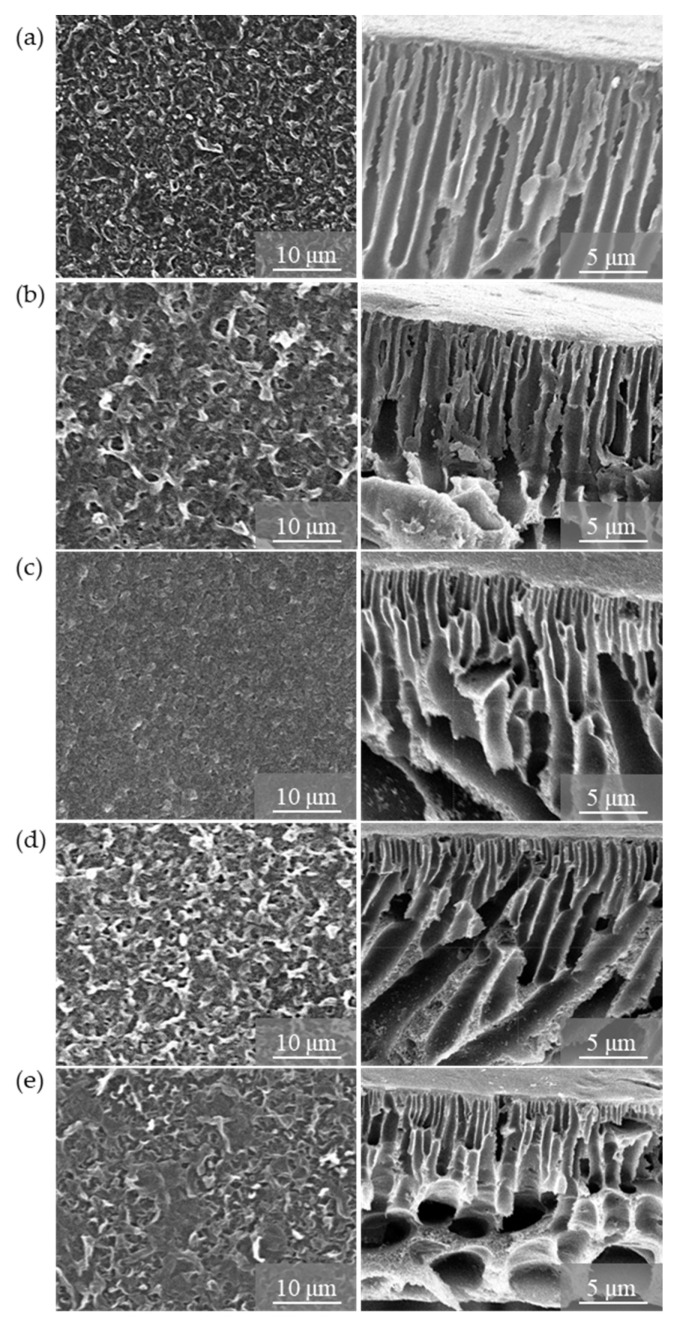
Top surface SEM images of TFN-GO membrane (left column) and cross section (right column): (**a**) TFN-0GO, (**b**) TFN-0.1GO, (**c**) TFN-0.25GO, (**d**) TFN-0.5GO and (**e**) TFN-1.0GO.

The top surface and cross-sectional morphology of TFN-GO membranes were observed as shown in Figure 6. According to Figure 6 (left column), all the PA active layers exhibited typical ridge-like-valley morphology. Detailed observation shows that with the increase of wt% of GO, the surface of the membrane becomes smoother, confirming the excellent dispersion of GO in the polymer matrix. However, when the wt% of GO increased from 0.5 to 1.0 wt%, the surface becomes rough. This was attributed to the reaction between MPD and TMC and with a hydrophilic group of GO; thus, it leads to a dense, compact, chain structure [48].

The cross-sectional images reveal the uniform, long, finger-like macro-voids in the TFC-0GO and TFC-0.1GO membranes. Meanwhile, for TFC-0.25GO and TFC-0.5GO, the larger finger-like macro-voids were not uniformly distributed and co-existed with a sponge-like structure in the bottom section of the membrane. However, as the GO loading increased further to 1.0 wt%, bigger, fine, oval-shaped pores were formed towards the bottom section of the membrane. A possible explanation of this phenomenon can be given based on the hydrophilic nature of GO. The hydrophilicity of GO could increase the thermodynamic incompatibility between the polymer and the solvent during the phase inversion process. The fast exchange rate process will lead to an extended porosity as well as changes in the structure of the macrovoids. However, with the addition of more GO (0.5 and 1.0 wt%), the sponge-like structure appeared as the higher viscosity of the dope solution decreased the phase separation rate which might otherwise have retarded the demixing process between the solvent and the non-solvent [17,23]. Hence, the morphology of TFN membranes was found to depend significantly on the amount of GO added. The digital photo images of the top and bottom layers of the TFN-GO membranes are shown in Figure S1 (see Supplementary Materials). It has been observed that the colour of the top layer exposed to water was darker than the bottom layer. The increasing wt% of GO will make the TFN-GO become darker and indicate that the GO is well dispersed in the polymer matrix.

**Figure 7:**
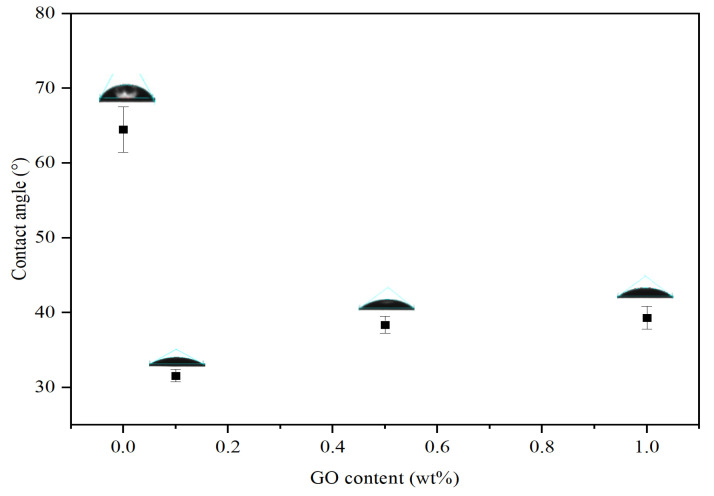
Water contact angle results for TFN-GO membranes.

The water CA result in Figure 7 could confirm the enhanced hydrophilicity of the TFN-GO membrane. The measurements of the CA were reproduced on ten samples and the error bar represents the standard deviation. The CA of pristine PSf membrane was measured at about 64° but was as low as 32° when GO was added. This change in CA is attributed to the excellent dispersion of GO in the polymer matrix, indicating that TFN-GO has an abundance of oxygenous functional groups such as hydroxyl, carboxyl and epoxy that has improved the hydrophilicity of TFN membrane significantly (see Figure 5). In addition, the GO functional groups present in the membrane surface will enhance the PA layer crosslinking, increasing the surface wettability of the membrane. The CA increased slightly to 39° when 1.0 wt% of GO was added. This result is consistent with previous studies that incorporated hydrophilic nanomaterials in the membrane substrate and showed decreases in their CA values [24,25].

### 3.3. Intrinsic Transport Properties, Porosity and Average Pore Sizes of TFN-GO Membranes

Table 1 lists the intrinsic transport properties of the TFN-GO membranes. The pure water permeability, *A*, was favourably enhanced when GO was introduced in the polymer matrix. Compared to TFN-0GO, TFN-0.25GO showed a four-fold increase in the value of *A*. It is known that the hydrophilicity of GO facilitates the solubilisation of water molecules at the membrane surface during the membrane filtration process, thus improving the water permeability [32]. However, the *A* values of TFN-0.5GO and TFN-1.0GO decreased. This is probably due to the non-uniform distribution of the higher wt% of GO added to the membrane substrate [49]. Hence, with respect to *A*, the addition of GO played a significant role in improving the performance of the TFN membrane.

TFN-0.25GO exhibited the highest salt rejection, *R* = 98.67%, which explains the lowest salt permeability, *B* = 0.24 Lm^−2^h^−1^. The value of *R* for all TFN membranes with added GO (0.1, 0.25, 0.5 and 1.0 wt%) improved compared to TFN-0GO. Furthermore, TFN-0.25GO exhibited the lowest *B/A* value, showing the highest separation efficiency; a low *B/A* value indicates a low salt reverse flux and high water permeability. Therefore, this is evidence that TFN-0.25GO possesses ideal and desirable intrinsic transport properties, among all the TFN membranes studied.

One of the vital factors influencing the performance of the TFN membranes is the *S* value, which is used to assess the degree of ICP effects of a membrane. As shown in Table 1, TFN-0GO shows the highest *S* value of 2712 μm, which indicates that the pristine PSf membrane is not suitable for FO-PRO processes. With the addition of GO into the membrane substrates, the *S* values decline sharply with TFN-0.25GO exhibiting the smallest *S* value, 726 μm. Generally, membranes incorporating GO have high *A* values and have lower *S* values, which is due to the improved hydrophilicity and reduced ICP effect [3].

**Figure 8:**
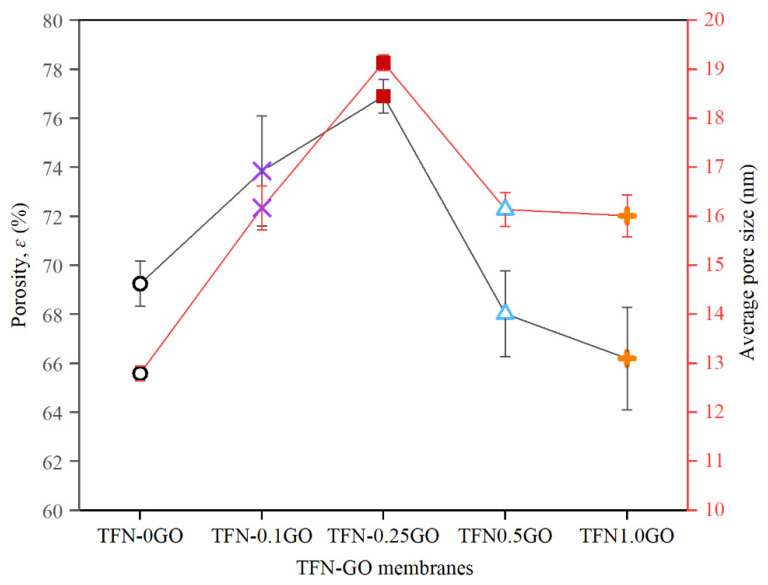
The porosity and mean pore size for TFN-GO membranes.

Figure 8 shows the overall porosity and the average pore size for all TFN-GO membranes studied. The measurements were measured on three samples and the error bar represents the standard deviation. The porosity initially increased from 69 ± 0.9% for TFN-0GO to 74 ± 2.2% and 77 ± 0.7% for TFN-0.1GO and TFN-0.25GO, respectively. This was credited to the high exchange rate of the solvent and the non-solvent (water) during phase inversion and resulted in the formation of large pores in the membrane. When the amount of GO was further increased to 1.0 wt%, the porosity reduced to 66 ± 2.0%. This was possibly due to the higher loading of agglomerated GO in the polymer matrix. The average pore size follows the porosity trend.

### 3.4. Forward Osmosis Performance

**Figure 9:**
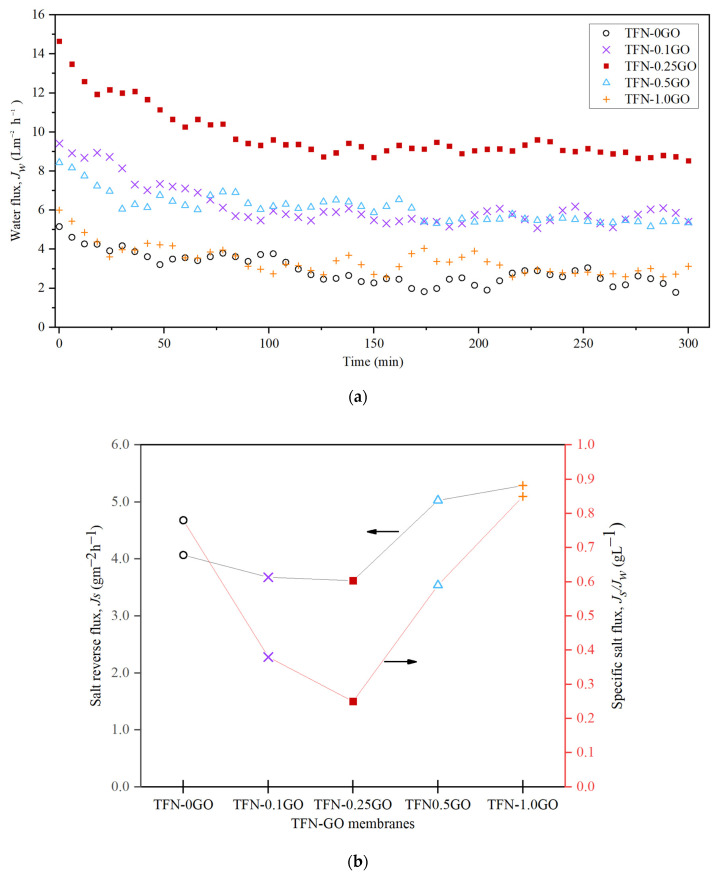
(**a**) Water flux trend and (**b**) salt reverse flux and specific salt flux under forward osmosis (FO) mode.

Figure 9 shows the performance of the TFN-GO membranes in FO mode using 1 M NaCl as the DS and DI water as the FS with the AL-FS membrane orientation. In general, membranes with additional GO exhibited higher *J_w_* compared to pristine PSf membrane. The *J_w_* improved remarkably from 5.15 Lm^−2^h^−1^ for TFN-0GO to 14.65 Lm^−2^h^−1^ for TFN-0.25GO. In addition, the trend of *J_w_* is relatively stable throughout the whole 300 min. These enhanced water fluxes are due to the improvement of their hydrophilicity, porosity and structural parameter as discussed in the previous section.

Meanwhile, the salt reverse flux, *J_s_*, also increased with GO content. The *J_s_* value is consistent with the value of *R* in the tests of the intrinsic transport properties of the membrane with the *J_s_* value of TFN-0.25GO measured as 3.62 gm^−2^h^−1^ compared to TFN-0GO, which was 4.07 gm^−2^h^−1^. Nonetheless, the TFN-0.5GO and TFN-1.0GO have slightly higher *J_s_* values of 5.03 gm^−2^h^−1^ and 5.29 gm^−2^h^−1^, respectively. This can be explained by the lessened crosslinking of the PA layer due to the larger opening structure of PA (Figure 6) that would reduce the salt rejection; hence, the *J_s_* value will be increased.

In addition, the specific salt flux, *J_s_/J_w_*, is an important parameter that can be used to determine the performance of the TFN membranes. In order to obtain a high performance FO membrane, low *J_s_/J_w_* and high *J_w_* are preferred. As shown in Figure 9b, the *J_s_/J_w_* value decreased from 0.78 gL^−1^ for TFN-0GO to 0.25 gL^−1^ for TFN-0.25GO. Likewise, the *J_s_/J_w_* increased for TFN-0.5GO and TFN-1.0GO because of the increase in the reverse salt flux. The excellent performance showed by TFN-0.25GO under FO mode was elucidated further under PRO mode, whether or not it can retain the same performance.

### 3.5. Pressure Retarded Osmosis Performance

The TFN-GO membranes were tested in PRO mode (AL-DS orientation) using 1 M NaCl as the DS and DI water as the FS. Pressures of 2–15 bar were applied in the draw solution side to examine the performance of the TFN-GO membranes under PRO. Generally, the thin film membrane under high pressure in PRO mode is very vulnerable to membrane deformations such as reduced membrane thickness, distortion of the PA layer and, ultimately, the membrane will burst [6,12,50]. Therefore, a non-woven spacer is used in this test to ensure the membranes can withstand pressure up to >15 bar before any sort of deformation occur to the membranes when passed a critical point. It was important to ensure the *W_ma_*_x_ for each membrane is achievable. The preliminary tests (see Figure S2) carried out without the non-woven spacer show that the TFN-0GO membrane can only endure a pressure up to 8 bar while the rest of the membranes, TFN-0.1GO, TFN-0.25GO, TFN-0.5GO and TFN-1.0GO were able to withstand pressure up to 11 bar before bursting.

**Figure 10:**
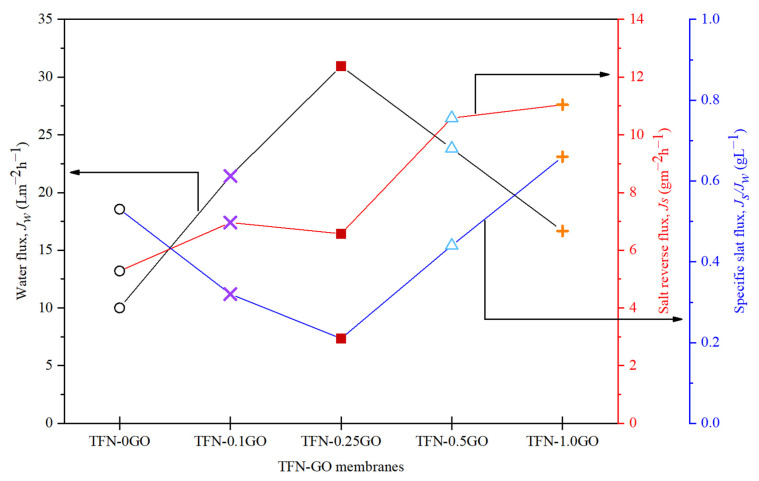
The optimum water flux, salt reverse flux and specific salt flux under PRO mode over a pressure range of 2–15 bar.

Figure 10 shows the *J_w_*, *J_s_* and *J_s_/J_w_* of the TFN-GO membranes with the highest *J_w_* (30.95 Lm^−2^h^−1^) and lowest *J_s_* achieved by TFN-0.25GO. This trend is consistent with the intrinsic transport properties as well as the FO performance. The highest *A* value and lowest *B* value together with smaller *S* values (see Table 1) will minimise the salt leakage and ICP effect and maintain the high effective driving force across the membrane [51]. The high *J_w_* of TFN-0.25GO was credited to the hydrophilic oxygen-bearing groups of GO as previously discussed. Meanwhile, the *J_w_* values of TFN-0.5GO and TFN-0.1GO also follow the previous test performance trends. As predicted, TFN-0GO showed the worst performance during the test with the lowest *J_w_* of 10 Lm^−2^h^−1^. The low porosity, higher *S* value and hydrophobic nature of PSf reduced the mass transfer coefficient, thus enhanced the ICP effect.

Interestingly, the *J_s_* value increases with the increase of GO loading in the TFN-GO membrane under high pressure. It was hypothesised that, when the applied pressure was increased, the flux decreased and the external concentration polarization (ECP) became less severe on the draw side of the membrane, resulting in the increase of salt crossover to the FS as the concentration of the DS against the membrane surface is higher. The salt crossover will also increase when membranes start to deform due to increased pressure [3]. Nevertheless, the *J_s_/J_w_* shows good stability for TFN-0.1GO and TFN-0.25GO, explaining how good performance could be maintained despite the increasing *J_s_* value. It is also consistent with the value of *R* (Table 1). When the applied pressure increases, the *J_s_/J_w_* is increased for TFN-0.5GO and TFN-1.0GO, indicating the possible initiation of the deformation of the selective layer of the membrane [51].

**Figure 11:**
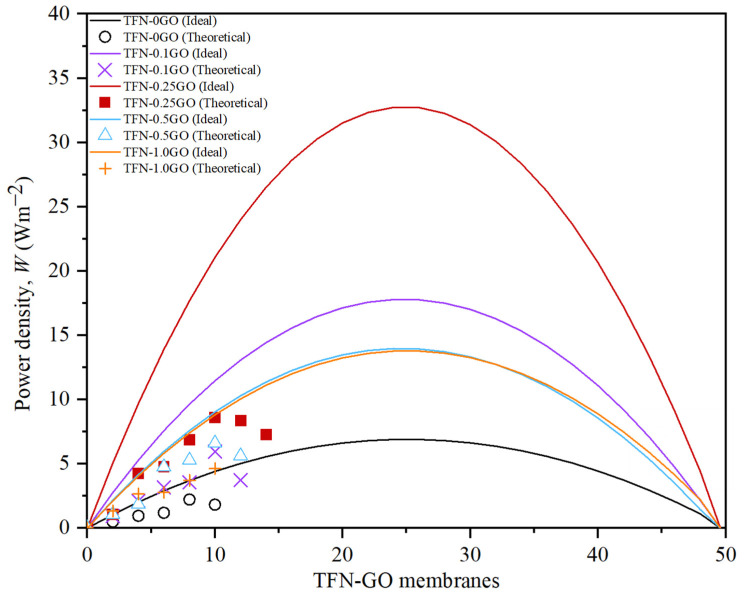
The theoretical and experimental results on the power density of TFN-GO membranes.

Another key performance indicator for PRO membranes is power density, *W*. Figure 11 presents the *W* achieved by TFN-GO membranes under high pressure (2–15 bar). All data were collected for 1 h for each pressure after 30 min for stabilisation after the pressure was increased. The figure compares the parabolic curve of the experimental *W* data (symbol) and the theoretical data (lines) (please see Figure S3 for individual graphs of both datasets). In theory, the optimal operating pressure is half of the osmotic pressure difference (ΔP = Δπ/2). The theoretical data applies to the ideal scenario of the PRO process with no consideration of ECP, ICP or salt reverse flux. Theoretical *W* parabolic curves for all TFN-GO membranes were computed with Equations (1)–(3) using the *A* values from Table 1. However, under actual experimental conditions, the effective osmotic driving force was greatly reduced due to the salt leakage, the ECP and the ICP that make the performance of TFN-GO membranes cannot achieve the theoretical maximum value.

At a pressure of 12 bar, the *J_w_* for TFN-0.25GO reached the highest value, resulting in the highest peak *W* at 8.36 Wm^−2^. Compared to TFN-0GO, the peak *W* of the TFN-0GO is 2.22 Wm^−2^ at a pressure of 8 bar. The low *A* value also resulted in the loss of driving force by hydraulic resistance. The results show that apart from *J_w_*, the *A* value is a vital parameter that influences *W_max_.* The peak *W* values of TFN-0.1GO, TFN-0.5GO and TFN-1.0GO were 5.95 Wm^−2^, 6.61 Wm^−2^ and 4.63 Wm^−2^, respectively. The comparison of these values with those previously reported in the literature is given in Table 2. The improvement of the TFN incorporated with GO in PRO performance clearly shows the hydrophilic nanomaterial is an efficient approach in producing high performance PRO membrane, in addition to its mechanical stability to the thin-film membrane.

As predicted, *W* decreases for all TFN-GO membranes when the pressure applied was >11 bar due to severe ICP that enhanced salt crossover and also possible deformation of the top of the membrane [11]. Those factors compromise the performance of the membrane but not to the extent the membrane is burst. The membrane was still intact without major damage after 5 h under pressures >15 bar. Of all the TFN membranes tested in this study, TFN-0.25GO gives excellent performance in PRO mode. Hence, the optimum GO loading of the polymer matrix is 0.25 wt%.

## 4. Conclusions

TFN-GO membranes have been successfully synthesised using a mixed matrix membrane using the NMP and IP processes to form the surface on top of the membrane. The introduction of GO into the polymer matrix has resulted in enhanced hydrophilicity, water flux, salt reverse flux, salt rejection and structural parameter. It has been observed that the addition of GO influenced the changes in membrane morphology of the TFN-GO membrane. The TFC-0.25GO not only achieved a power density of 8.36 Wm^−2^ but can also withstand an applied pressure > 15 bar. However, with further increase in the GO wt% added to the polymer matrix, GO agglomeration may occur, leading to lowered performance of the membrane and deformation under high pressure. Hence, 0.25 wt% is the optimum addition to the polymer matrix for the improvement of pressure retarded osmosis membranes.

## Figures and Tables

**Table 1:** The intrinsic transport properties of TFN-GO membranes.

Membrane	Water Permeability, *A* (Lm^−2^h^−1^bar^−1^)	Salt Permeability, *B* (Lm^−2^h^−1^)	*B/A* (bar)	Salt Rejection, *R* (%)	Structural Parameter, *S* (μm)
TFN-0GO	0.40 ± 0.25	0.70 ± 0.02	1.75	89.2 ± 0.15	2712
TFN-0.1GO	1.04 ± 0.33	0.37 ± 0.01	0.36	96.23 ± 0.08	1012
TFN-0.25GO	1.91 ± 0.34	0.24 ± 0.02	0.13	98.67 ± 0.13	726
TFN-0.5GO	0.82 ± 0.38	0.48 ± 0.01	0.59	93.96 ± 0.06	1027
TFN-1.0GO	0.80 ± 0.06	0.68 ± 0.01	0.85	91.50 ± 0.13	1659

**Table 2:** The performance of PRO membranes under different conditions.

Membrane	Feed Solution	Draw Solution	Operation Pressure (bar)	Water Flux (Lm^−2^h^−1^)	Power Density (Wm^−2^)	Reference
TFN-0.1GO	DI water	1 M NaCl	10	21.43	5.95	Current study
TFN-0.25GO	DI water	1 M NaCl	12	30.95	8.36	Current study
TFC-PAN	DI Water	0.5 NaCl	12.07	28.2	8	[12]
Polyamide TFC-PRO	DI water	1 M NaCl	15	40 * − 90 ± 2	7–12	[46]
Porifera, commercial FO	DI water	58.44 g/L NaCl	10.4	28.8	7.1	[52]
HTI, commercial asymmetric CTA	DI water	60 g/L NaCl	9.72	18.76	5.06	[53]
TFC-PEI-CNT	DI Water	1 M NaCl	~22	23.2	15	[11]

* estimated from the graph.

## Supplementary Materials

The following are available online at https://www.mdpi.com/2077-0375/10/12/416/s1, Figure S1: Digital picture of TFN-GO membrane (a) top surface (b) bottom surface. From left TFN-0GO, TFN0.1GO, TFN0.25GO, TFN-0.5GO and TFN-1.0GO. Figure S2: Burst point (membrane damaged) power density of TFN-0GO and TFN-0.25GO. Figure S3: The power density graph of all the TFN-GO membranes.
