# Supplementary material for: Graphene Oxide Incorporated Polysulfone Substrate for Flat Sheet Thin Film Nanocomposite Pressure Retarded Osmosis Membrane
Source: Membranes (Basel). 2020 Dec 11;10(12):416. doi: 10.3390/membranes10120416 (PMC7763650; doi:10.3390/membranes10120416)
Supplement: Supplementary file 1 [file membranes-10-00416-s001.pdf]

Supplementary Material

S1

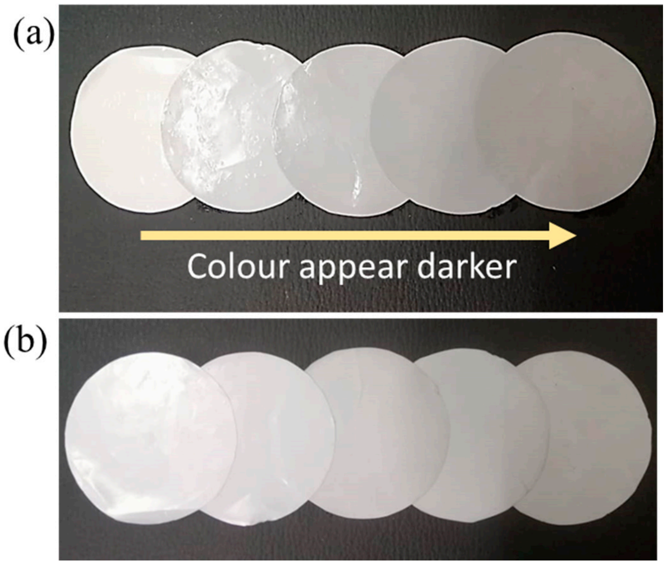

Figure S1. Digital picture of TFN-GO membrane (a) top surface (b) bottom surface. From left TFN-0GO, TFN0.1GO, TFN0.25GO, TFN-0.5GO and TFN-1.0GO.

S2

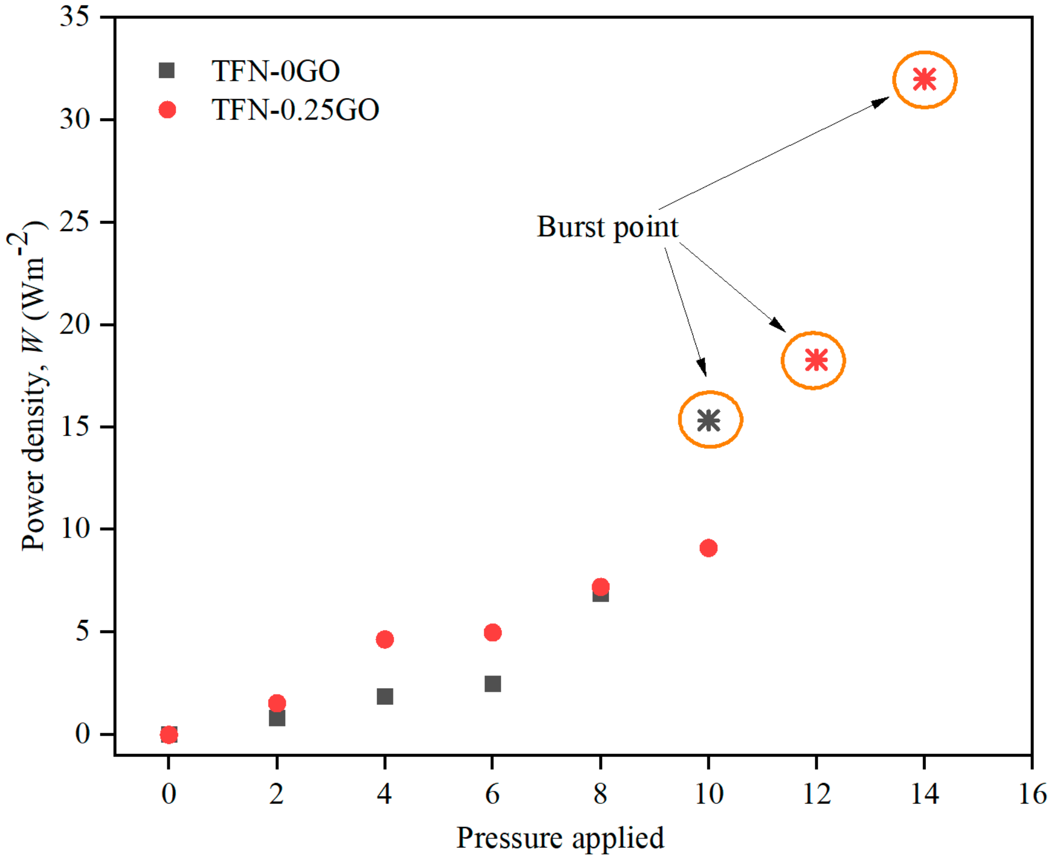

Figure S2. Burst point (membrane damaged) power density of TFN-0GO and TFN-0.25GO.

S3

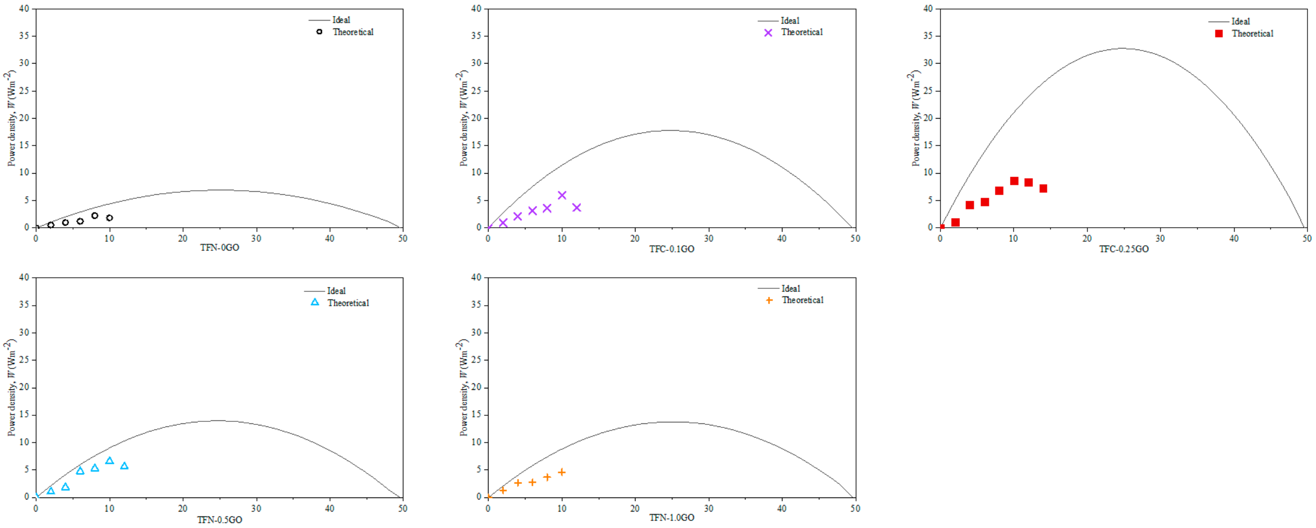

Figure S3. The power density graph of all the TFN-GO membranes.
